# Brainwave viscosity in propofol anaesthesia

**DOI:** 10.1016/j.bja.2021.11.005

**Published:** 2022-02

**Authors:** M.S. Fabus, M.W. Woolrich, C.E. Warnaby

**Affiliations:** Wellcome Centre for Integrative Neuroimaging, University of Oxford, Oxford, UK

## Abstract

Human EEG during propofol anaesthesia shows large-scale changes including traveling slow waves^1^. Slow-wave saturation is a potentially individualised marker of loss of perception^2^. However, much remains unclear about the dynamics of slow waves. Iterated empirical mode decomposition (itEMD^3^) is a novel data-driven method for segregating data into physiologically relevant oscillatory modes. We used itEMD to identify spectral modes and their sources / sinks in propofol EEG. Viscosity is a physical quantity expressing the magnitude of resistance to flow. Considering traveling electric potentials in the brain as a flow, we extended the notion of viscosity to traveling brainwaves. Using this, we explored how brainwave viscosity changes in volunteer propofol anaesthesia.

Data came from an ultra-slow propofol infusion to 4 μg ml^-1^ in 16 healthy volunteers^2^. 32-channel EEG referenced to FCz was acquired. Data was re-referenced to linked earlobes, down-sampled to 200Hz, bandpass filtered 0.1-30Hz, and decomposed using itEMD. Instantaneous amplitude from each channel was found using the Hilbert transform, interpolated onto a 15 × 15 scalp grid, and its velocity field calculated. Normalised velocity was computed for the data and for a surrogate dataset with a pre-motor 2Hz dipole. Timepoints with significant flow (*P*<0.01 against surrogate) were analysed. Singularities in the flow were identified as extremal contours in the velocity divergence. Multiplying area and divergence of sinks/sources, $V=A\nabla \cdot \vec{𝜈}\left(\left[{\text{m}}^{2}{\text{s}}^{-1}\right]\right)$ gave brainwave viscosity. Induction was divided into three equal concentration bins. Median viscosity across singularities for each level was extracted for each subject. Group-level differences were tested using a Bonferroni-corrected paired t-test. Research was funded in part by the Wellcome Trust (203139/Z/16/Z, 106183/Z/14/Z, 215573/Z/19/Z) and the MRC Development Pathway Funding Scheme (MR/R006423/1). For the purpose of open access, the authors have applied a CC-BY public copyright licence to any Author Accepted Manuscript version arising from this submission.

Three low-frequency modes with different properties were identified in the data: High Delta (~4Hz), Low Delta (~2Hz), Slow (<1Hz). Waves originated from frontally dominant sources and ended in posteriorly dominant sinks. Brainwave viscosity was lowest for slow waves (*P*<10^-4^) and decreased at high propofol doses (*P*<10^-3^, Figure 1).

We used a novel spectral decomposition method to identify important brain modes in propofol anaesthesia. Extending the concept of viscosity, we found brainwave diffusion is frequency- and concentration-dependent. This potentially intrinsic property of the brain decreased in deep propofol sedation and was lowest for slow waves. We hypothesise this may be due to network changes in the anaesthetised brain resulting in easier slow-wave spread.

## Figures and Tables

**Figure 1:**
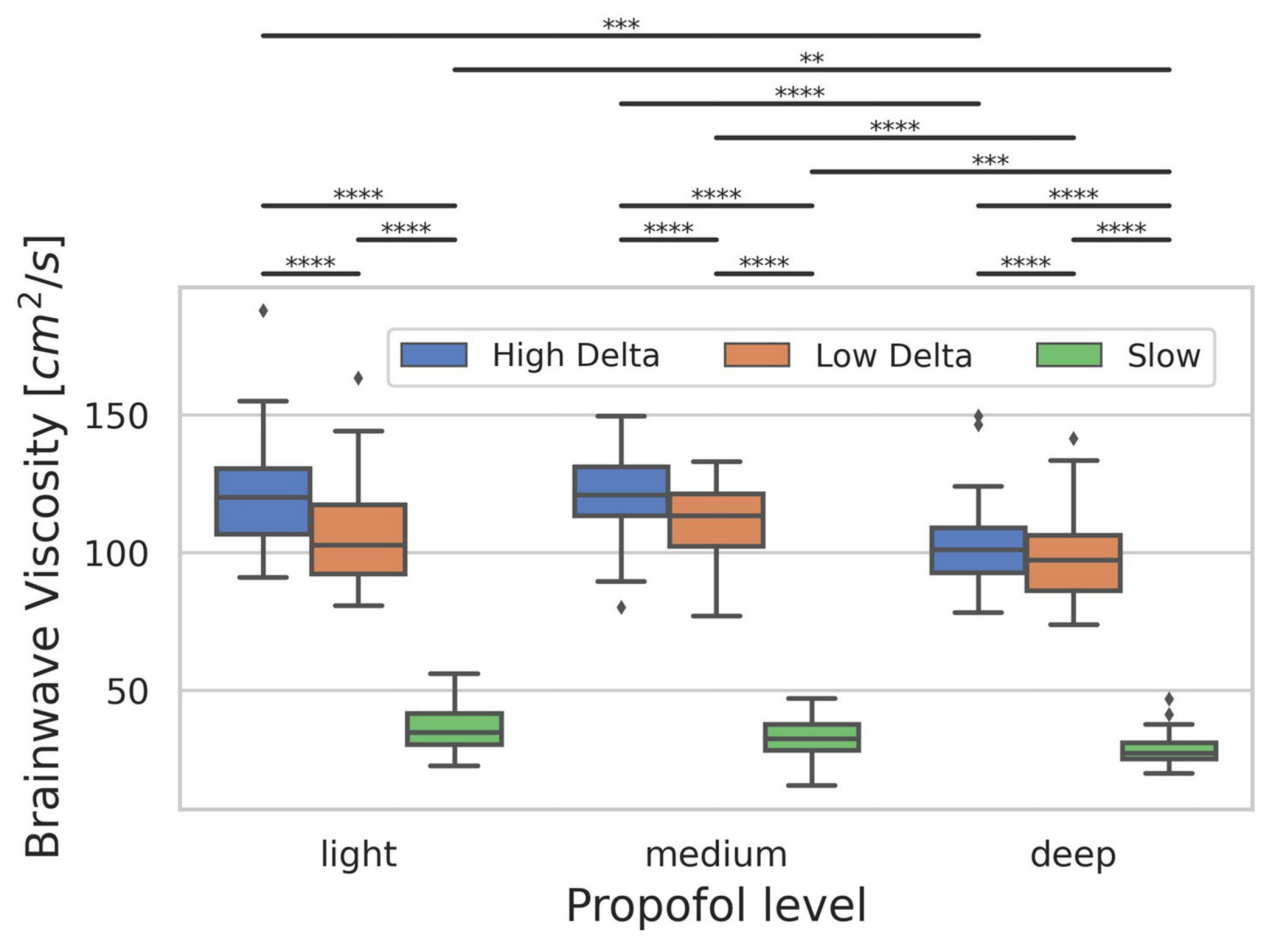
Brainwave viscosity in propofol anaesthesia. Only significant comparisons are shown with Bonferroni-corrected P<10^-N^ shown as N stars.
